# Prevalence and risk factors for atrial fibrillation in dogs with myxomatous mitral valve disease

**DOI:** 10.1111/jvim.15927

**Published:** 2020-10-08

**Authors:** Carlo Guglielmini, Marlos Goncalves Sousa, Marco Baron Toaldo, Carlotta Valente, Vinicius Bentivoglio, Chiara Mazzoldi, Ilaria Bergamin, Michele Drigo, Helen Poser

**Affiliations:** ^1^ Department of Animal Medicine, Production and Health University of Padua Padua Italy; ^2^ Department of Veterinary Medicine Federal University of Paraná Curitiba Brazil; ^3^ Department of Veterinary Medical Sciences University of Bologna Bologna Italy

**Keywords:** canine, cardiac arrhythmia, echocardiography, electrocardiography, epidemiology

## Abstract

**Background:**

Atrial fibrillation (AF) is a common supraventricular arrhythmia more frequently observed in large breed dogs.

**Objectives:**

Estimate the prevalence of AF in dogs with myxomatous mitral valve disease (MMVD) and identify risk factors for developing AF.

**Animals:**

A total of 2194 client‐owned dogs with MMVD, including 1280, 588, 290, and 36 dogs in ACVIM stage**s** B1, B2, C, and D, respectively.

**Methods:**

Retrospective, cross‐sectional study. The medical databases of 3 veterinary teaching hospitals were reviewed. Inclusion criteria were a diagnosis of MMVD after complete cardiovascular evaluation and cardiac rhythm assessment using routine 2‐minute ECG or good quality ECG tracing during echocardiographic examination.

**Results:**

Atrial fibrillation was diagnosed in 59 dogs with a prevalence of 2.7%. Univariate analysis showed that mixed breed, male sex, advanced ACVIM stage, left atrial and ventricular enlargement, fractional shortening (FS), and presence of pulmonary hypertension were significantly associated with development of AF. According to 2 multivariable models, the left atrium (LA)‐to‐aorta ratio (odds ratio [OR] = 14.011, 7.463‐26.304), early trans‐mitral velocity (OR = 2.204, 1.192‐4.076), body weight (OR = 1.094, 1.058‐1.130), and FS (OR = 0.899, 0.865‐0.934) and LA (OR = 5.28, 3.377‐8.092), advanced ACVIM stage (OR = 4.922, 1.481‐16.353), and FS (OR = 0.919, 0.881‐0.959) were significant predictors of AF for models 1 and 2, respectively.

**Conclusions and Clinical Importance:**

Atrial fibrillation is an uncommon complication of MMVD and is significantly associated with the more advanced stage of the disease, increased LA dimension and body weight, and decreased FS.

**Abbreviations**
2D: 2‐dimensional
ACVIM: American College of Veterinary Internal Medicine
AF: atrial fibrillation
Ao: aortic root diameter
AUC: area under the curve
BW: body weight
CI: confidence interval
CT: cardiac treatment
E max: mitral valve maximal E wave velocity
FS: fractional shortening
HF: heart failure
LA: left atrial diameter
LA/Ao: left atrium to aorta ratio
LAE: left atrial enlargement
LV: left ventricle
LVDD: left ventricular diastolic diameter
LVDDn: left ventricular diastolic diameter normalized for body weight
LVSD: left ventricular systolic diameter
LVSDn: left ventricular systolic diameter normalized for body weight
MMVD: myxomatous mitral valve disease
MT: mixed treatment
NPV: negative predictive value
NT: no treatment
OR: odds ratio
OT: other treatments
PH: pulmonary hypertension
PPV: positive predictive value
ROC: receiver operating characteristic
TR: tricuspid regurgitation
VTH: veterinary teaching hospital

## 1 INTRODUCTION

Atrial fibrillation (AF) is the most common supraventricular arrhythmia in both humans and dogs.^1^, ^2^, ^3^, ^4^ Although some dogs can develop AF in the absence of recognizable cardiac disease (ie, primary or lone AF), AF secondary to cardiac diseases associated with left atrial enlargement (LAE) is more commonly observed.^3^, ^4^ In humans, the prevalence and risk factors associated with development of AF are well known.^1^, ^2^, ^5^, ^6^, ^7^ In particular, heart failure (HF) and AF are linked by similar risk factors in people and share a common pathophysiology.^1^, ^2^, ^5^, ^6^, ^7^ Thus, it is widely accepted that HF and AF can cause and exacerbate each other through mechanisms such as structural cardiac remodeling, activation of neuro‐hormonal mechanisms, and rate‐related impairment of left ventricular function.^1^, ^2^, ^5^, ^6^, ^7^

In dogs, AF usually is observed in animals with dilated cardiomyopathy, myxomatous mitral valve disease (MMVD), or congenital cardiac disease associated with LAE.^3^, ^4^ Among these diseases, MMVD is the most frequently diagnosed, accounting for approximately 70% of acquired cardiac disease in dogs.^8^, ^9^, ^10^ Progressive LAE is a common sequela during MMVD progression, and guidelines provided by the American College of Veterinary Internal Medicine (ACVIM) established a specific classification scheme of the different stages of HF associated with MMVD.^9^, ^10^ Although some epidemiologic data and associations for AF development have been described in dogs,^3^, ^11^ no study has thoroughly examined the prevalence rate and risk factors for this arrhythmia in dogs with MMVD. In addition, results of a recent study showed that the presence of AF is associated with a worse prognosis in medium to large‐sized dogs with MMVD.^12^ Thus, knowledge of the risk factors for developing AF is useful in the clinical evaluation of dogs with MMVD.

We estimated the prevalence of AF in a large population of dogs with MMVD and identified the risk factors for developing AF in these animals. We hypothesized that AF is associated with worsening of MMVD and that some clinical variables and echocardiographic parameters indicative of cardiac remodeling can be useful predictors of developing AF in dogs with MMVD.

## 2 MATERIAL AND METHODS

### 2.1 Animals

For this retrospective study, data were collected from the internal database of the veterinary teaching hospitals (VTH) of the Universities of Padova, Bologna, and Curitiba. Dogs were recruited to the study from those undergoing cardiac diagnostic evaluation and final diagnosis of MMVD between January 2012 and December 2018. Diagnosis of MMVD was based on clinical and echocardiographic findings including a left apical systolic murmur, thickened or prolapsing mitral valve leaflets or both on 2‐dimensional (2D) echocardiography associated with mitral valve regurgitation on color flow Doppler interrogation.^9^, ^10^ Disease severity was classified according to the ACVIM guidelines.^9^, ^10^ The presence or absence of AF was based on at least 1 of the following methods: routine surface ECG recording with dedicated machines of at least 2 minutes duration or good quality ECG recordings during the echocardiographic examination. In particular, the echocardiographic diagnosis of AF was based on the combined presence of the following findings: irregularly irregular cardiac rhythm with narrow QRS complexes, isoelectric trace without recognizable P waves, and absence of A wave on mitral inflow on Doppler interrogation.

For dogs with more visits available during the period of observation, only data obtained during the most recent visit and those obtained during the first visit documenting AF were analyzed for dogs without AF and those developing AF, respectively. Dogs with sinus rhythm and dogs with cardiac arrhythmias other than AF were merged together and only 2 groups were considered for analysis: dogs with AF and dogs without AF. The absence of a final rhythm diagnosis was considered an exclusion criterion. Dogs with equivocal cardiac diagnosis or other concomitant congenital or acquired cardiac disease also were excluded from this study.

### 2.2 Echocardiographic examination

At each VTH, an experienced operator performed the echocardiographic examination. The left ventricular diameter at diastole (LVDD) and systole (LVSD) were measured from M‐mode short‐axis echocardiographic images at the level of the chordae tendinae and fractional shortening (FS) then was calculated according to the formula (LVDD‐LVSD)/LVDD. Normalized left ventricular (LV) dimensions (ie, LVDSn and LVDDn) were calculated according to reported allometric scaling.^13^ Left atrial diameter (LA) and aortic root diameter (Ao) were measured at early diastole from 2D echocardiographic short axis images obtained at the level of the heart base and the LA/Ao ratio then was calculated.^14^, ^15^ Pulsed‐wave Doppler interrogation of trans‐mitral blood flow was obtained from the left apical 4‐chamber view and the peak of the early diastolic wave (E max) was measured. Multiple views were used to evaluate trans‐tricuspid blood flow and the maximal value of any tricuspid regurgitation (TR) velocity was measured using continuous‐wave Doppler. In the absence of right ventricular outflow obstruction, the presence of pulmonary hypertension (PH) was considered when the TR maximal velocity was ≥3 m/s, corresponding to a Doppler‐derived estimated systolic pressure gradient of 36 mm Hg.^16^

### 2.3 Statistical analysis

Data were entered into an electronic spreadsheet (Microsoft Excel, Microsoft Office 2011, Microsoft Corporation, Bellevue, Washington). Statistical analyses were carried out using commercially available software (IBM Corp. Released 2016. IBM SPSS Statistics for Windows, Version 24.0. Armonk, New York: IBM Corp.; MedCalc Statistical Software version 16.4.3, MedCalc Software, Ostend, Belgium). Data obtained from the case records were: sex, breed, age, body weight (BW), ongoing treatment at the time of examination, and ACVIM class. For breed, ACVIM class, and ongoing treatment, the following binary or quaternary categories were considered, respectively: purebred and crossbred; dogs with compensated MMVD (ie, dogs with MMVD but without past or current development of HF, corresponding to ACVIM classes B1 and B2) and dogs with decompensated MMVD (ie, dogs with MMVD with past or current evidence of HF, corresponding to ACVIM classes C and D)^9^, ^10^; dogs receiving no treatment (NT), any treatment for cardiac disease (CT), any other treatment for noncardiac disease (OT), and mixed treatment (MT) for concurrent cardiac and noncardiac disease, at time of enrollment. The following continuous echocardiographic variables were considered: LA, LA/Ao, LVDDn, LVSDn, FS, and E max. Furthermore, LA/Ao ≥ 1.6, LVDDn ≥ 1.7, LVSDn ≥ 1.2, FS ≥ 40%, E max ≥ 1.2 m/s, and TR velocity ≥ 3 m/s were used to create the binary variables classifying dogs with or without LAE, LV diastolic and systolic enlargement, hyperdynamic LV, increased LA pressure, and PH, respectively.^10^, ^13^, ^14^, ^15^, ^16^, ^17^

Continuous data were assessed graphically for normality and presented as mean and SD, whereas categorical variables are presented as number and percentage within each category. Comparison of clinical and echocardiographic variables between dogs with MMVD developing AF and those not developing AF was carried out using the Student's *t* test and the Fisher's exact test or the Chi‐square test with Yate's correction for continuous and categorical variables, respectively.

Ability to distinguish between dogs developing AF and those not developing AF of the main continuous variables showing a significant difference between dogs with and without AF (ie, BW, LA, LA/Ao, LVDDn, LVSDn, FS, and E max) was evaluated by receiver operating characteristic (ROC) curve analysis. The corresponding area under the curve (AUC) with binomial exact confidence interval (CI) was calculated successively. In particular, the sensitivity, specificity, and negative (NPV) and positive predictive values (PPV) were calculated at various cut‐off points.

Univariate and multivariable logistic regression analysis estimated the odds ratio (OR) with 95% CI of developing AF. In particular, the following variables were analyzed as dichotomous variables and assessed in the univariate analysis: breed (crossbred vs purebred), sex (female vs male), BW (≤20 kg vs >20 kg), LA (<3.45 cm vs ≥3.45 cm), LA/Ao (<1.6 vs ≥1.6), LVDDn (<1.7 vs ≥1.7), LVSDn (<1.2 vs ≥1.2), FS (<40% vs ≥40%), E‐max (<1.2 m/s vs ≥1.2 m/s), PH (yes vs no), treatment (OT vs CT + MT + NT), and ACVIM stage (B1 + B2 vs C + D). Two multivariable models then were built using variables significantly associated with AF in the univariate analysis after evaluation of autocorrelation and interaction between predictors. After applying the stepwise backward elimination method, model 1 included BW and the echocardiographic predictors LA/Ao, E‐max, and FS as continuous variables, and model 2 included the echocardiographic predictors LA and FS as continuous variables, and ACVIM stage as a dichotomous categorical variable. The Exp(B) was calculated and reported as OR with 95% CI for each incremental unit or for the level of interest of the continuous variables and the categorical variable, respectively.

A value of *P* < .05 was considered significant.

## 3 RESULTS

### 3.1 Study population and echocardiographic parameters

A total of 2194 dogs with MMVD were recruited in the study, including 1280, 588, 290, and 36 dogs in the ACVIM stages B1, B2, C, and D, respectively. Pulmonary hypertension was diagnosed in 526 dogs (23.9%). At enrollment, 1628 dogs (74.2%) were receiving NT; 311 dogs (14.2%) ≥1 drugs for cardiac disease; 47 dogs ≥1 drugs for both cardiac disease and concomitant noncardiac disease (2.1%); and 208 dogs (9.5%) ≥1 drugs only for concomitant noncardiac disease. Supplemenatry Appendix shows the complete list of the various drugs administered to dogs belonging to each treatment group. Most of the dogs were crossbreds (851 dogs, 38.8%), and Miniature and Toy Poodle (280 dogs, 12.8%), Miniature Pinscher (130 dogs, 5.9%), Dachshund (115 dogs, 5.2%), Lhasa Apso (106 dogs, 4.8%), Yorkshire terrier (74 dogs, 3.4%), Cocker spaniel (70 dogs, 3.2%), and Cavalier King Charles Spaniel (51 dogs, 2.3%) were the most commonly represented pure breeds. There were 1159 (52.8%) females. The mean age was 136 months (SD = 36 months) and the mean BW was 10.9 kg (SD = 8.5 kg).

Atrial fibrillation was diagnosed in 59 dogs with a prevalence of 2.7%. Twenty of these cases (33.9%) were diagnoses by an ECG tracing obtained during the echocardiographic examination. Table 1 presents a comparison of clinical and echocardiographic variables between dogs with AF and those without AF. In dogs with AF, the percentage of crossbred dogs, male dogs, animals with PH, dogs with decompensated HF, and dogs receiving CT was significantly higher when compared to that of purebred dogs (*P* = .002), female dogs (*P* < .001), dogs without PH (*P* < .001), dogs with compensated HF (*P* < .001), and dogs receiving NT, MT, or OT (*P* < .001, *P* = .007, and *P* < .001), respectively. Among the continuous variables, dogs with AF had significantly increased mean BW, heart rate, LA, Ao, LA/Ao, LVDDn, LVDSDn, and E max (*P* < .001 for all comparisons), and significantly decreased mean FS (*P* < .001) compared to dogs without AF, whereas mean age was not different (*P* = .69).

**TABLE 1:** Descriptive data obtained from 2194 dogs with myxomatous mitral valve disease with and without atrial fibrillation (AF)

Variable	Category	AF group, n = 59 dogs		No AF group, n = 2135 dogs		*P* value
		Number (%), mean ± SD	Missing values, n (%)	Number (%), mean ± SD	Missing values, n (%)	
Breed	Crossbred	35 (59.3)		817 (38.3)		.002
	Purebred	24 (40.7)		1318 (61.7)		
Age (mo.)	mean ± SD	136 ± 28		136 ± 35	27 (1.3)	.69
Sex	Female	18 (30.5)		1141 (53.5)	3 (0.1)	.001
	Male	41 (69.5)		991 (46.5)		
BW (kg)	Mean ± SD	17.3 ± 10.6		10.7 ± 8.6	11 (0.5)	<.001
HR (bpm)	Mean ± SD	187 ± 41	1 (1.7)	128 ± 31	5 (0.2)	<.001
LA (cm)	Mean ± SD	4.98 ± 0.97	1 (1.7)	2.38 ± 0.80	2 (0.1)	<.001
Ao (cm)	Mean ± SD	1.91 ± 0.48	1 (1.7)	1.60 ± 0.43	2 (0.1)	<.001
LA/Ao	Mean ± SD	2.72 ± 0.67	1 (1.7)	1.52 ± 0.47	2 (0.1)	<.001
LVDDn	Mean ± SD	2.06 ± 0.36		1.55 ± 0.32	20 (0.9)	<.001
LVSDn	Mean ± SD	1.26 ± 0.28		0.85 ± 0.23	22 (1.0)	<.001
FS (%)	Mean ± SD	36.9 ± 10.4		43.5 ± 9.6	11 (0.5)	<.001
E max (m/s)	Mean ± SD	1.49 ± 0.37	3 (5.1)	0.83 ± 0.36	19 (0.9)	<.001
PH	No	31 (52.5)		1638 (76.7)		<.001
	Yes	28 (47.5)		497 (23.3)		
ACVIM stage	B1 + B2	6 (10.2)		1861 (87.2)		<.001
	C + D	53 (89.8)		274 (12.8)		
**Treatment**	CT/NT	48 (84.8)/9 (15.8)		263 (14.0)/1619 (86.0)		<.001
	CT/MT	48 (100.0)/0 (0.0)		263 (84.8)/47 (15.2)		.007
	CT/OT	48 (96.0)/2 (4.0)		263 (56.1)/206 (43.9)		<.001
	MT/OT	0 (0.0)/2 (100.0)		47 (18.6)/206 (81.4)		.81
	MT/NT	0 (0.0)/9 (100.0)		47 (2.8)/1619 (97.2)		.62
	NT/OT	11 (84.6)/2 (15.4)		206 (11.3)/1619 (88.7)		.81

Abbreviations: ACVIM, American College of Veterinary Internal Medicine; AF, atrial fibrillation; BW, body weight; CT, cardiac treatment; E max, mitral valve maximal E wave velocity; FS, fractional shortening; HR, heart rate; LA, left atrial diameter; LA/Ao, left atrial diameter to aortic diameter; LVDDn, left ventricular diastolic diameter normalized to body weight; LVSDn, left ventricular systolic diameter normalized to body weight; MT, mixed treatment; n, number of dogs; NT, no treatment; OT, other treatments; PH, pulmonary hypertension.

Table 2 presents the results of the ROC curve analyses. The LA (AUC = 0.979; 95% CI, 0.972‐0.984) and LA/Ao (AUC = 0.931; 95% CI, 0.920‐0.941) had high accuracy to predict development of AF at the cut offs >3.45 cm and >1.8, respectively. Both echocardiographic indices had very high sensitivity (98.3% for both variables) and high specificity (89.8% and 78.5% for LA and LA/Ao, respectively) to predict the presence of AF. In particular, LA > 3.45 cm had a PPV of 20.7% and NPV of 99.9% to predict development of AF. The BW, LVDDn, LVSDn, and E max were only moderately accurate to predict development of AF (AUC = 0.735; 95% CI, 0.716‐0.754; 0.854, 0.796‐0.912; 0.875, 0.821‐0.929; 0.900, 0.887‐0.912, respectively).

**TABLE 2:** Diagnostic accuracy of 7 clinical and echocardiographic variables to predict developing of atrial fibrillation in 2194 dogs with myxomatous mitral valve disease

Variable	AUC	95% CI	Cut‐off	Sensitivity (%)	Specificity (%)	PPV (%)	NPV (%)
BW (kg)	0.735	0.716‐0.754	7.6	96.6	44.4	4.6	99.8
LA (cm)	0.979	0.972‐0.984	>3.45	98.3	89.8	20.7	99.9
LA/Ao	0.931	0.920‐0.941	>1.8	98.3	78.5	11.0	99.9
LVDDn	0.854	0.796‐0.912	>1.82	81.4	82.2	10.1	99.4
LVSDn	0.875	0.821‐0.929	>1.08	79.7	86.7	14.4	99.3
FS (%)	0.682	0.662‐0.702	≤40.1	70.7	60.1	4.7	98.7
E max (m/s)	0.900	0.887‐0.912	>1.02	91.1	79.0	10.3	99.7

Abbreviations: AUC, area under the curve; BW, body weight; CI, confidence interval; E max, mitral valve maximal E wave velocity; FS, fractional shortening; LA, left atrial diameter; LA/Ao, left atrial diameter to aortic diameter; LVDDn, left ventricular diastolic diameter normalized to body weight; LVSDn, left ventricular systolic diameter normalized to body weight; NPV, negative predictive value; PPV, positive predictive value.

### 3.2 Univariate and multivariable analysis

Univariate logistic regression showed that developing AF was positively correlated with mixed breeds (*P* = .002), male sex, BW ≥20 kg, LA ≥3.45 cm, LA/Ao ≥1.6, LVDDn ≥1.7, E max ≥1.2 m/s, FS < 40%, presence of PH, and ACVIM stage C + D (*P* < .001 for all variables; Table 3).

**TABLE 3:** Results of the univariate logistic regression analysis showing the association between the risks for developing atrial fibrillation in 2194 dogs with myxomatous mitral valve disease

Variable	Category	Odds ratio (95% CI)	*P* value
Breed	Crossbred	0.425 (0.251‐0.720)	.002
	Purebred		
Sex	Female	2.623 (1.497‐4.594)	.001
	Male		
BW (kg)	1‐20	2.61 (1.44‐4.70)	<.001
	>20		
LA (cm)	<3.45	499.793 (68.891‐3625.909)	<.001
	≥3.45		
LA/Ao	<1.6	134.768 (18.621‐975.370)	<.001
	≥1.6		
LVDDn	<1.7	13.508 (6.796‐26.847)	<.001
	≥1.7		
LVSDn	<1.2	4.744 (0.548‐41.049)	.22
	≥1.2		
FS (%)	<40	0.303 (0.176‐0.522)	<.001
	≥40		
E max (m/s)	<1.2	26.28 (13.44‐51.41)	<.001
	≥1.2		
PH	No	2.977 (1.768‐5.011)	<.001
	Yes		
**Treatment**	OT	0.33 (0.04‐1.26)	.163
	CT + NT + MT		
ACVIM stage	B1 + B2	59.99 (25.55‐140.89)	<.001
	C + D		

Abbreviations: ACVIM, American College of Veterinary Internal Medicine; BW, body weight; CT, cardiac treatment; E max, mitral valve maximal E wave velocity; FS, Fractional shortening; LA, left atrial diameter; LA/Ao, left atrial diameter to aortic diameter; LVDDn, left ventricular diastolic diameter normalized to body weight; LVSDn, left ventricular systolic diameter normalized to body weight; MT, mixed treatment; NT, no treatment; OT, other treatments; PH, pulmonary hypertension.

Two final multivariable models were built including only significant predictors and accounting for multicollinearity (Table 4). In model 1, increased LA/Ao was confirmed as a strong risk factor for developing AF (OR = 14.011; 95% CI, 7.463‐26.304; *P* < .001) followed by E max and BW (OR = 2.204 and 1.094; 95% CI, 1.192‐4.076 and 1.058‐1.130; *P* = .003 and *P* < .001, respectively). Decreased FS also was associated with the risk of developing AF (OR = 0.899; 95% CI, 0.865‐0.934; *P* < .001). Model 2 showed that increased LA and decompensated ACVIM stage are risk factors for developing AF (OR = 5.280 and 4.922; 95% CI, 3.377‐8.092 and 1.481‐16.353; *P* < .001 and *P* = .009, respectively) and confirmed the effect of decreased FS (OR = 0.919; 95% CI, 0.881‐0.959; *P* < .001).

**TABLE 4:** Results of the multivariable logistic regression analysis for the risks for developing atrial fibrillation in 2194 dogs with myxomatous mitral valve disease

Predictors	Odds ratio	Lower 95% CI	Upper 95% CI	*P* value
*Model 1*				
LA/Ao	14.011	7.463	26.304	<.001
E‐max	2.204	1.192	4.076	.003
BW	1.094	1.058	1.130	<.001
FS	0.899	0.865	0.934	<.001
*Model 2*				
LA	5.280	3.377	8.092	<.001
ACVIM stage C + D	4.922	1.481	16.353	.009
FS	0.919	0.881	0.959	<.001

Abbreviations: ACVIM, American College of Veterinary Internal Medicine; BW, body weight; E max, mitral valve maximal E wave velocity; FS, fractional shortening; LA, left atrial diameter; LA/Ao, left atrial diameter to aortic diameter.

## 4 DISCUSSION

The main results of our study were that development of AF is uncommonly associated with MMVD in dogs, accounting for 2.7% of cases. Increased BW, LA, LA/Ao, and E max, and decreased FS as well as decompensated stage of HF were associated with a higher risk for developing AF in these dogs.

A previous epidemiological study conducted from 1969 to 2007 using the Veterinary Medical Data Base that included 26 colleges of veterinary medicine in the United States found an increase of AF diagnosis in dogs over the study period ranging from 5.07 to 23.31 per 10 000 canine admission without any information regarding underlying cardiac diseases.^11^ Large differences also were found according to breed and associated BW because the prevalence of AF was 5.84% and 0.04% in Irish Wolfhounds and Miniature Poodles, respectively.^11^ On the other hand, some studies specifically evaluated cardiac rhythm in dogs with MMVD. In 1 study, a longitudinal evaluation using standard ECG of 257 dogs found AF in 1.6% of them.^18^ Three other studies evaluated cardiac rhythm using standard ECG or Holter monitoring or both in 36 dogs with MMVD of different stages, 43 dogs with advanced MMVD with or without syncope, and 90 dogs with compensated and decompensated MMVD.^19^, ^20^, ^21^ The presence of AF was only found in 4 dogs (11.1%) of the former study, all of them in symptomatic stages of the disease.^19^ Furthermore, in a study that compared clinical findings in dogs with MMVD, including 58 German Shepherds dogs and 49 small breed dogs (ie, BW < 15 kg), AF was more prevalent in the former group when compared to latter dogs.^22^ Our study included a large group of dogs of different breeds, representative of the entire population of dogs affected by MMVD regarding either the signalment, because small breed dogs with advanced age were overrepresented, or different disease stage and complications (eg, percentage of dogs with Doppler‐derived diagnosis of PH).^8^, ^9^, ^10^, ^16^, ^23^ The prevalence rate of 2.7% found in our study suggests that AF is an uncommon complication of MMVD. Precise epidemiological data seldom are available in the veterinary medical literature. In addition to the direct useful information it conveys, knowledge of prevalence rates of a given disease allows calculation of the PPV and NPV of a diagnostic test or clinical variable. These predictive values are more intuitive compared to the more commonly reported sensitivity and sensibility and are clinically very useful, as discussed below.^24^

Dogs with MMVD that developed AF had many differences in clinical and echocardiographic variables compared to dogs without AF. In particular, AF was more prevalent in crossbred, male dogs with higher BW. Interestingly, age was similar in both groups of dogs (ie, mean age > 11 years for both groups), which represents an important difference when compared to humans where advanced age is a risk factor for developing AF.^1^, ^2^, ^5^, ^6^, ^7^ Regarding echocardiographic variables, dogs with AF had significantly increased LAE (ie, significant increases of both LA and LA/Ao), LV dimension (both in systole and diastole), and E max that represent an indirect index of increased left atrial pressure,^25^ and significantly decreased FS. Unsurprisingly, AF was found in a significantly higher percentage of dogs with decompensated HF as well as in those with PH likely because these changes are associated with a more advanced stage of MMVD.^9^, ^10^, ^17^, ^25^, ^26^, ^27^ Regarding evaluation of the diagnostic accuracy for the above‐mentioned continuous variables to predict development of AF, BW, LVDDn, LVSDn, FS, and E max were only moderately accurate, but LA and LA/Ao had high accuracy to predict development of AF at the cut‐offs of 3.45 cm and 1.8, respectively. In particular, these 2 variables had excellent sensitivity (ie, 98.3% for both variables) and high specificity (ie, 89.8% and 78.5%, respectively). However, because of the observed low prevalence rates of AF in dogs with MMVD, only LA reached a clinically useful PPV of 20.7%. This observation means that among dogs with MMVD that have LA > 3.45 cm, it can be predicted that only 1 in 5 actually will develop AF.

Univariate analysis showed that crossbred, male dogs with increased BW, LA, LA/Ao, LVDDn, and E max, decreased FS and with PH and decompensated HF had increased risk of developing AF. However, after the multivariable analyses, only the following variables remained significantly associated with increased risk of developing AF: increased BW, LA/Ao, LA, and E max, decreased FS, and decompensated HF. These findings suggest that left atrial remodeling, characterized by both LAE and increased LA pressure, is a major risk factor for developing AF in dogs with MMVD. In particular, per each cm and an increment unit of LA and LA/Ao, there is a 5‐ and 14‐fold increase in risk of developing AF, respectively. Similarly, for each 1 m/s increase in E max, there is a 2‐fold increased risk of developing AF. Body weight and decompensated HF are additional risk factors, meaning that for each kilogram of increased BW there is a 9% increased risk, and dogs with ACVIM stages C and D have 5‐fold increased risk of developing AF. Conversely, for each 1% decrease of FS, there is an 8% to 10% increased risk of developing AF. Many factors can be responsible for decreased FS in dogs with MMVD with or without AF including higher BW, advanced stage of disease, loss of the atrial component of LV filling, and tachycardia‐induced cardiomyopathy.^12^, ^22^, ^28^, ^29^, ^30^

Atrial structural, electrical, ionic, and functional remodeling changes are considered the fundamental pathophysiological mechanisms underlying AF in humans.^31^, ^32^ In particular, left atrial structural remodeling refers to adaptive or maladaptive changes in cardiac architecture that occur at the macro‐ and microscopic levels.^31^, ^32^ The hallmark of macroscopic change is atrial dilatation, whereas fibrosis is the most important microscopic change.^31^, ^32^ In our study, the observed association of increased LA and LA/Ao as well as increased E max emphasizes the importance of left atrial remodeling as a risk factor for developing AF in dogs with MMVD. Body weight was an additional risk factor for developing AF in dogs, as compared with humans in whom obesity and increased body mass index but not BW were risk factors identified for developing AF.^33^ This difference between humans and dogs can be easily explained by the large breed‐associated variability in the BW of dogs. Furthermore, BW also has an effect on LAE. Because absolute left atrial dimension depends on BW and severity of cardiac disease leading to LAE, not surprisingly LA had the highest diagnostic accuracy and PPV to predict AF, and also compared to the LA/Ao, which represents only a relative index of LAE. Atrial fibrosis is 1 of the most important abnormalities contributing to the development of AF in humans.^34^ Potential mediators known to promote fibrosis in the atrium include pressure and volume overload, aging, atrial stretch, inflammation, and oxidative stress.^34^ As atrial myopathy progresses, it can lead to atrial dysfunction.^34^ Few studies have investigated the microscopic changes of atrial myocytes in dogs with MMVD or AF or both.^35^, ^36^, ^37^ In dogs with MMVD, the observed changes included myocardial fatty replacement, immune cell infiltration, and interstitial fibrosis,^35^, ^36^ whereas atrial histological changes in dogs with AF were investigated mainly in animals with experimentally induced arrhythmias.^37^ Evaluation of left atrial function recently has been investigated in dogs with MMVD using speckle‐tracking echocardiography, and the results suggested the presence of left atrial dysfunction in more advanced stages of the disease.^38^, ^39^, ^40^, ^41^, ^42^ Furthermore, in addition to absolute cardiac diameters, evaluation of LA function with speckle‐tracking echocardiography can be useful to predict development of AF in dogs with MMVD.^43^

Decompensated HF is a risk factor for AF in dogs with an OR (4.9) similar to that observed in humans (4.5‐5.9) in the classical Framingham heart study.^44^ Heart failure and AF are linked by similar risk factors in humans and share a common pathophysiology. Thus, it is widely accepted that HF and AF can cause and exacerbate each other through mechanisms such as structural cardiac remodeling, activation of neuro‐hormonal mechanisms, and rate‐related impairment of LV function.^44^ Our results and those of another recent study suggest that the same relationship between AF and HF also could be present in dogs.^29^ However, the precise cause‐effect relationship between HF and AF remain elusive in our study as well as in studies of humans,^45^ because the temporal relationship of each condition could not be determined. In addition, the prevalence of AF in HF is known to increase with increased severity of pump failure in humans.^46^ Evaluation of the systolic function is challenging in dogs with MMVD and was not specifically addressed in our study. However, the observed risk factor of decreased FS suggests that a similar pathophysiological mechanism also might be present in dogs, but results of FS measurements in subjects with MMVD can be misleading.^28^

Our study had some limitations because of its retrospective design. The cardiac rhythm evaluation relied either on ECG recording using a dedicated machine, the reference method for cardiac rhythm evaluation, or using a good quality ECG recording during echocardiographic examination. This latter represents a less reliable method of accurate cardiac rhythm analysis and approximately one‐third of AF cases were diagnosed using echocardiography. However, because the aim of our study was to identify dogs with AF compared to dogs with any other cardiac rhythm, we propose that AF has specific ECG characteristics (ie, irregularly irregular cardiac rhythm with narrow QRS complexes and isoelectric trace without recognizable P wave) and modifications of cardiac function (eg, absence of the A wave during trans‐mitral Doppler interrogation) that are easily recognizable during echocardiographic examinations. Evaluation of the left atrial dimension was based on linear echocardiographic measurements whereas other more accurate methods of left atrial size measurements, such as an estimation of left atrial volume using the Simpson disk method or use of 3D echocardiography,^47^, ^48^ were not performed. However, measurement of the LA and calculation of LA/Ao are the more commonly employed echocardiographic methods of evaluating left atrial size in a clinical setting. Only a limited number of clinical variables were retrieved, and therefore we could not evaluate the effect of some risk factors for developing AF in humans such as obesity, systemic hypertension, and other comorbidities (eg, diabetes mellitus, chronic kidney disease).^1^, ^2^ However, systemic hypertension does not seem to be an important issue in dogs with MMVD, even in the most advanced stage of the disease.^49^ In addition, the number of available echocardiographic variables was limited. Although the presence of other cardiac diseases was an exclusion criterion, we could not completely rule out the presence of subtle cardiac diseases (eg, arrhythmogenic cardiomyopathy) in the early phase of their progression. No thorough information regarding specific concomitant extra‐cardiac diseases could be retrieved, but the administration of OT, a surrogate of other relevant concomitant diseases, neither showed any statistical difference in dogs with AF nor represented a risk factor of AF development. Finally, AF is not a unique entity but can be classified as paroxysmal, persistent, long‐standing persistent, and permanent according to the duration of arrhythmic episodes.^1^, ^2^ This scheme of classification described in humans is seldom used in veterinary medicine, and no attempt was made in our study to better define the specific type of AF.

## 5 CONCLUSIONS

In conclusion, AF is uncommonly associated with AF in dogs with MMVD. Left atrial remodeling associated with LAE and increased left atrial pressure, increased BW, decreased FS, and decompensated HF are risk factors for developing AF in dogs. Conversely, increased age, a demonstrated risk factor of developing AF in humans, does not appear to play a similar role in dogs. Future studies investigating the complex pathophysiological mechanisms of this arrhythmia should include further evaluation of atrial pathology and dysfunction in dogs with AF.

## CONFLICT OF INTEREST DECLARATION

Authors declare no conflict of interest.

## OFF‐LABEL ANTIMICROBIAL DECLARATION

Authors declare no off‐label use of antimicrobials.

## INSTITUTIONAL ANIMAL CARE AND USE COMMITTEE (IACUC) OR OTHER APPROVAL DECLARATION

Authors declare no IACUC or other approval was needed.

## HUMAN ETHICS APPROVAL DECLARATION

Authors declare human ethics approval was not needed for this study.

## Supporting information


**Appendix** **S1**: Supplementary Information.
