# Supplementary material for: Prevalence and risk factors for atrial fibrillation in dogs with myxomatous mitral valve disease
Source: J Vet Intern Med. 2020 Oct 8;34(6):2223–31. doi: 10.1111/jvim.15927 (PMC7694843; doi:10.1111/jvim.15927)
Supplement: Supplementary file 1 — Appendix S1: Supplementary Information. [file JVIM-34-2223-s001.pdf]

Appendix 1: Type of treatment and drugs administered to 2,194 dogs with myxomatous mitral valve disease with and without atrial fibrillation (AF)

Therapy / Drugs	AF Group, n = 59 dogs<br>Number (%)	No AF Group, n = 2135 dogs<br>Number (%)	Total, n= 2194 dogs<br>Number (%)
**Cardiac treatment**	**48 (15.4)[a]**	**263 (84.6)[a]**	**311 (100)[b]**
ACE-Is	45 (17.4)	214 (82.6)	259 (83.3)
Pimobendan	29 (18.1)	131 (81.9)	160 (51.4)
Furosemide-Torasemide	41 (21.5)	150 (78.5)	191 (61.4)
Digoxin	17 (85)	3 (15)	20 (6.4)
Spironolactone	16 (23.2)	53 (76.8)	69 (22.2)
Sildenafil	6 (24)	19 (76)	25 (8.0)
Hydroclortiazide	5 (71.4)	2 (28.6)	7 (2.3)
Amlodipine	4 (28.6)	10 (71.4)	14 (4.5)
Diltiazem	3 (100)	0 (0)	3 (1.0)
Sotalol	1 (100)	0 (0)	1 (0.3)
Mexiletine	1 (100)	0 (0)	1 (0.3)
Amiodarone	0 (0)	1 (100)	1 (0.3)
**Mixed treatment**	**0 (0)**	**47 (100)**	**47 (100)**
ACE-Is	0 (0)	35 (100)	35 (74.5)
Pimobendan	0 (0)	11 (100)	11 (23.4)
Furosemide-Torasemide	0 (0)	10 (100)	10 (21.3)
Spironolactone	0 (0)	16 (100)	16 (34)
Amlodipine	0 (0)	5 (100)	5 (10.6)
Sildenafil	0 (0)	3 (100)	3 (6.4)
Antibiotics	0 (0)	10 (100)	10 (21.3)
Steroids	0 (0)	5 (100)	5 (10.6)
NSAIDs	0 (0)	4 (100)	4 (8.5)
Anticonvulsants	0 (0)	3 (100)	3 (6.4)
Antacids-Antiemetics	0 (0)	2 (100)	2 (4.3)
Antihistamines	0 (0)	2 (100)	2 (4.3)
Trilostane	0 (0)	10 (100)	10 (21.3)
Levothyroxine	0 (0)	2 (100)	2 (4.3)
Insulin	0 (0)	2 (100)	2 (4.3)
Chemotherapy	0 (0)	1 (100)	1 (2.1)
Chondroitin sulphate	0 (0)	10 (100)	10 (21.3)
Opioids	0 (0)	5 (100)	5 (10.6)
Hepatoprotective drugs	0 (0)	4 (100)	4 (8.5)
**Other treatments**	**2 (1.0)**	**206 (99.0)**	**208 (100)**
Antibiotics	2 (3.0)	64 (97.0)	66 (31.7)
Steroids	0 (0)	26 (100)	26 (12.5)
NSAIDs	0 (0)	33 (100)	33 (15.9)
Anticonvulsants	0 (0)	47 (100)	47 (22.6)
Antacids-Antiemetics	0 (0)	49 (100)	49 (23.6)
Antihistamines	0 (0)	6 (100)	6 (2.9)
Trilostane	0 (0)	21 (100)	21 (10.1)
Levothyroxine	0 (0)	4 (100)	4 (1.9)

Insulin	0 (0)	13 (100)	13 (6.3)
Chemotherapy	0 (0)	6 (100)	6 (2.9)
Chondroitin sulphate	0 (0)	19 (100)	19 (9.1)
Opioids	0 (0)	15 (100)	15 (7.2)
Hepatoprotective drugs	0 (0)	10 (100)	10 (4.8)
Vitamins-Other supplements	0 (0)	6 (100)	6 (2.9)
Lipid lowering	0 (0)	3 (100)	3 (1.4)
Xanthine	0 (0)	3 (100)	3 (1.4)
Acetyl cysteine	0 (0)	2 (100)	2 (1.0)
Salbutamol	0 (0)	1 (100)	1 (0.5)
Ketoconazole	0 (0)	1 (100)	1 (0.5)
Antiandrogen	0 (0)	1 (100)	1 (0.5)
Tranexamic acid	0 (0)	1 (100)	1 (0.5)
Clopidogrel	0 (0)	2 (100)	2 (1.0)
**No therapy**	**9 (0.6)**	**1619 (99.4)**	**1628 (100)**

[a]Within treatment type (drug) percentages (row); [b]within treatment group percentages (column)

ACE-Is: angiotensin-converting enzyme inhibitors; NSAIDs: non-steroidal anti-inflammatory drugs.
